# Bioactive Compounds and Antioxidant Activity in Different Grafted Varieties of Bell Pepper

**DOI:** 10.3390/antiox4020427

**Published:** 2015-06-23

**Authors:** Celia Chávez-Mendoza, Esteban Sanchez, Ezequiel Muñoz-Marquez, Juan Pedro Sida-Arreola, Maria Antonia Flores-Cordova

**Affiliations:** Coordinación en Tecnología de Productos Hortofrutícolas y Lácteos Centro de Investigación en Alimentación y Desarrollo A C, Avenida Cuarta Sur No. 3820 Fraccionamiento Vencedores del Desierto. Cd. Delicias, Chihuahua C.P. 33089, Mexico; E-Mails: celia.chavez@ciad.mx (C.C.-M.); emunoz@ciad.mx (E.M.-M.); johnvih@gmail.com (J.P.S.-A.); mariflor_556@hotmail.com (M.A.F.-C.)

**Keywords:** bell pepper, bioactive compounds, grafting

## Abstract

Grafting favors the presence of bioactive compounds in the bell pepper, but many species and varieties have not yet been analyzed in this sense, including commonly grafted varieties. The aim of the present study is to characterize the content in β-carotenes, vitamin C, lycopene, total phenols, and the antioxidant activity of bell pepper (*Capsicum annum* L) using the cultivar/rootstock combinations: Jeanette/Terrano (yellow), Sweet/Robusto (green), Fascinato/Robusto (red), Orangela/Terrano (orange), and Fascinato/Terrano (red). The plants were grown in a net-shading system and harvested on three sampling dates of the same crop cycle. The results show statistical differences (*p* ≤ 0.05) between cultivar/rootstock combinations and sampling dates for the content in bioactive compounds and antioxidant activity. Fascinato/Robusto presented the highest concentration of lycopene and total phenols as well as the greatest antioxidant activity of all cultivar/rootstock combinations evaluated. In addition, it was found that the best sampling time for the peppers to have the highest concentrations of bioactive compounds and antioxidant activity was September.

## 1. Introduction

Currently, people have interest in maintaining good health and an excellent body figure, therefore, they have become more careful in the food they choose to consume, looking for food with a high nutritional value, bioactive compounds and antioxidant capacity, such as fruits and vegetables.

Epidemiological studies have consistently demonstrated a positive relation between the consumption of fruits and vegetables and a reduction in the mortality rate due to heart disease, cancer, and other degenerative diseases, as well as aging. This is attributed to the fact that these foods are the main source of nutraceutical compounds, such as vitamins, minerals, and phenolic compounds, natural antioxidants, fiber, and other biotic compounds [1,2].

The bell pepper (*Capsicum annum* L) is a fruit well known for its high content in bioactive compounds and strong antioxidant capacity and it is among the most popular of fresh vegetables worldwide due to its combination of color, flavor, and nutritional value [3]. The plant, native to North and South America, is most productive in warm, dry climates and is used both medicinally as well as a food in Africa and other countries of the world [4].

Currently, a broad number of varieties are available in the supermarkets, most of which change from a green color to yellow, orange, red, or purple when they are completely ripe. Green peppers are often harvested before they completely ripen, and the maturity stage can partly account for the content in phytonutrients and thus the consumption of antioxidants in the diet [5].

Fresh peppers have exceptionally high quantities of ascorbic acid and their attractive red color is due to several carotenoid pigments that include β-carotene with pro-vitamin A activity and oxygenated carotenoids such as capsantine, capsorubin, and cryptocapsin, which are exclusive to these fruits and have proven to be effective at scavenging free radicals [6]. Peppers also contain large quantities of neutral phenolic compounds or flavonoids called quercetin, luteolin, and capsaicinoids [7].The consumption of these bioactive compounds provide beneficial effects in human health due to their antioxidant properties, which protect against the oxidative damage to cells and thus prevent the development of common degenerative diseases such as cancer, cardiovascular diseases, cataracts, diabetes, Alzheimer’s, and Parkinson’s [3]. These chemical compounds also prevent the oxidation of essential fats within the cells of the brain that are considered necessary for its optimal functioning [8].

Due to the economic and nutritional importance of bell peppers, actually the use of grafting is increasing in this crop, the main applications have been to control nematodes, soil-borne diseases [9,10], and wilt caused by *Phytophthora capsici* [11,12,13]. In addition, grafting has been evaluated for its effect on the crop yield and morphological characteristics of the fruit [14], moreover, grafting favors the presence of certain phytochemical compounds and antioxidant activity, but many species and varieties still have not been analyzed in this sense, including commonly grafted varieties.

The aim of the present study is to characterize the content in bioactive compounds and antioxidant activity of different varieties of grafted bell pepper (*Capsicum annum* L) harvested on three different sampling dates in the same crop cycle.

## 2. Experimental Section

### 2.1. Approach and Experimental Design

For the experiment, the commercial varieties of bell pepper (Syngenta Seed, Houston, TX, USA) Jeanette, Sweet, Fascinato and Orangela were used as scions and were grafted from either Terrano or Robusto rootstock (Syngenta Seeds).

The fruits of grafted bell pepper were analyzed in order to determine the content in bioactive compounds and antioxidant activity. The pepper fruits were obtained from a commercial crop belonging to an agricultural company that exports this produce grown under net shading. In the experiment, the following bell pepper cultivar/rootstock combinations (Syngenta Seeds) were studied and used as treatments: (1) Jeanette/Terrano (yellow); (2) Sweet/Robusto (green); (3) Fascinato/Robusto (red); (4) Orangela/Terrano (orange); and (5) Fascinato/Terrano (red). The commercial rootstocks Terrano and Robusto were used due to their resistance to the wilt caused by the fungus *Phytophthora capsici* and to nematodes, in addition to their excellent affinity for the main pepper varieties to which they provide a vigorous root system and enable balanced vegetative development, favoring a healthy plant structure [15].

Rootstock seeds were sown in December 2012 and the varieties seeds were sown in January 2013. Commercial varieties were grafted 31 days after sowing. Thirty-seven days after the grafting, the plants were transplanted to beds under net shading. The average temperature was 32 °C and relative humidity of 52%. The plants were grown in a loamy sandy-clayey soil (57.36% sand, 12.08% silt, and 29.84% clay) with 1.68% organic matter and whose chemical characteristics are: pH 7.72, electrical conductivity of 0.84 dS/m, 50.17 ppm inorganic-N, 64.14 ppm phosphorus and CIC of 32.5 me/100 g.

The fertilization program in kg per hectare for a cycle of 240 days was applied with the following forms and rates: Fert 05–30 (49.5 mL/m^2^); phosphonitrate (34.3 g/m^2^); potassium sulfate (25.2 g/m^2^); ammonium nitrate (92.2 g/m^2^); calcium nitrate (46.8 mL/m2); potassium nitrate (26.3 g/m^2^); magnesium sulfate (85.6 g/m^2^); urea (0.8 g/m^2^) super K (14.2 mL/m^2^); flussing 8–24 (33.5 mL/m^2^); supplemented with commercial fertilizers.

The fruits harvested were classified as commercial quality according to the standard México Calidad Suprema [16].

For this study, three samples were made in the same 2013 crop cycle, the first on 4 July, the second on 20 August, and the third on 17 September.

One lot from each treatment was evaluated and, from each lot, fruits were sampled from 10 plants, these being considered as the experimental unit. The fruits were cleaned and taken to the laboratory to measure the content in vitamin C, total phenols, lycopene, β-carotene, and antioxidant capacity.

### 2.2. Determination of Bioactive Compounds

#### 2.2.1. Vitamin C

Vitamin C content was determined by HPLC as described by Doner and Hicks [17]. The analysis was done with fresh bell pepper. The pepper pulp was cut into small pieces and ground to mash using a kitchen blender. 10 g of bell pepper were then placed in 20 mL of an extraction mixture. Then, it was homogenized and filtered before being analyzed. The sample was injected into a Varian HPLC ProStar 320 equipped with a UV-Vis detector (ProStar 210, Prostar Varian Inc., Walnut Greek, CA, USA). An amine column Varian of 10 cm and a 20 μL injection loop were used. The analysis was done in triplicate.

**Figure 1:**
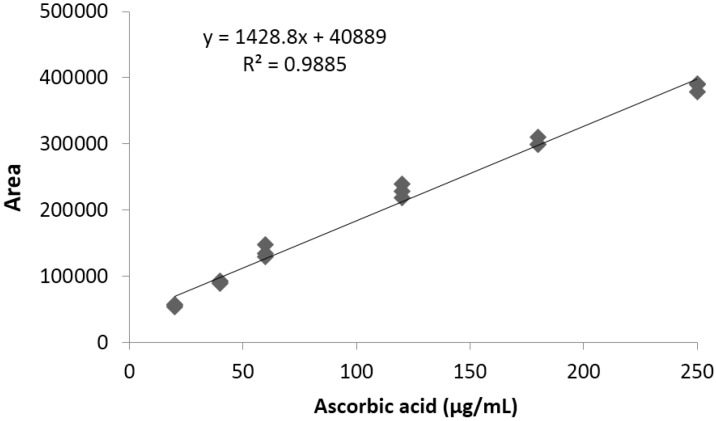
Representative standard curve obtained for vitamin C.

The limit of detection and quantification of the analytical method used for vitamin C quantification were 1.3 and 3.9 μg/mL, respectively. Linearity was determined between 0 and 250 μg/mL, using a standard ascorbic acid (vitamin C) high purity reagent grade, calibrating in triplicate. A typical calibration curve obtained for vitamin C is illustrated in Figure 1.

#### 2.2.2. Total Phenols

The phenolic compounds of bell pepper were extracted with methanol. Total phenolic content was quantified by A_765_ with Folin-Ciocalteau reagent as described by Singleton and Rossi [18]. The limit of quantification of the analytical method used for total phenols quantification were 1.38 and 4.19 μg/mL, respectively. The analysis was done in triplicate.

**Figure 2:**
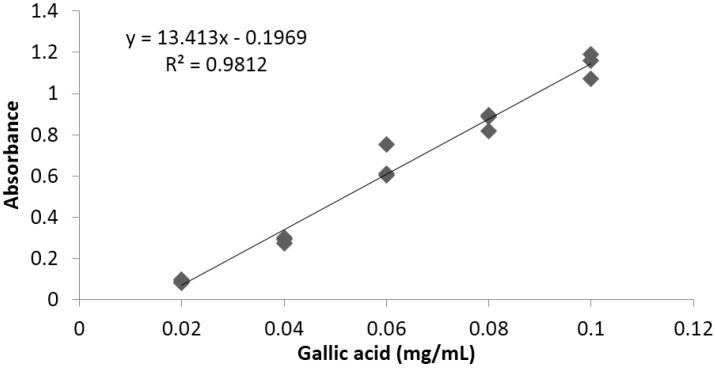
Representative standard curve obtained for total phenols.

Linearity was determined between 0.02 and 0.1 μg/mL, using a standard gallic acid high purity reagent grade, calibrated in triplicate. A typical calibration curve obtained for phenols is illustrated in Figure 2.

#### 2.2.3. Lycopene

The lycopene content of bell pepper was determined according to the methodology reported by Cucu *et al.* [19]. Before analysis, the fresh bell pepper was milled, then, 1 g of pepper was mixed for 1 min with 20 mL of extraction buffer solution (hexane/acetone/ethanol 2:1:1) using an Ultra-Turrax on ice. The obtained mixture was filtered and transferred into a separatory funnel, 50 mL of saturated NaCl solution was added and the solution was mixed for 1 min; the formation of two phases was allowed and once phases were well-separated, the aqueous phase was discarded and the hexane phase recovered and filtered over anhydrous NaSO_4_ (5 g) which was rinsed twice with 2.5 mL of extraction buffer solution. The filtrate was dried under nitrogen and the resulting residue was redissolved in tetrahydrofuran (THF) with butylated hydroxytoluene (BHT), and triethylamide (TEA) at 0.05% to appropriate concentrations before being injected.

Identification and quantification of lycopene was carried out by HPLC, using a Varian ProStar 320 HPLC system with UV-Vis ProStar 210 detector (Prostar Varian Inc., Walnut Greek, CA, USA) at 472 nm and a reversed-phase C18 column of 10 cm with MeOH/isopropyl alcohol/THF (30:30:35) containing 250 ppm BHT and 0.05% TEA as the mobile phase. Flow rate was 1 mL/min, the temperature of the column was 35 °C, and the injection volume was 20 μL.

Standard solutions of lycopene were prepared and a calibration curve was obtained. Lycopene was identified by comparing the retention time with the reference standard and the quantification was done by external standard calibration based on peak area. The total lycopene content was quantified as the sum of the areas of the peaks of all the E and Z isomers, and based on the standard curve of all E-lycopene. The analysis was done in triplicate.

**Figure 3:**
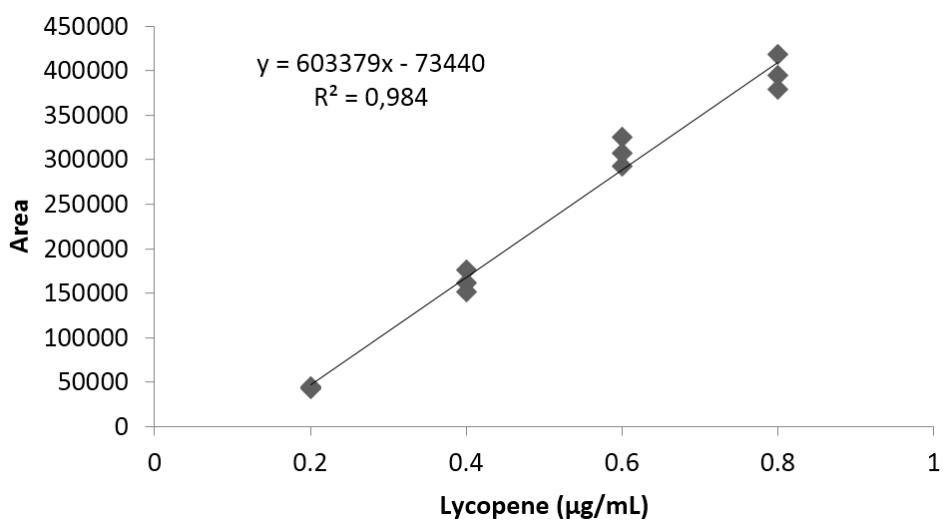
Representative standard curve obtained for lycopene.

The limit of detection and limit of quantification of the analytical method used for lycopene quantification were 0.01 and 0.02 μg/mL, respectively. Linearity was determined between 0.2 and 0.8 μg/mL, using a standard lycopene high purity reagent grade, calibrating in triplicate. A typical calibration curve obtained for lycopene is illustrated in Figure 3.

#### 2.2.4. β-Carotene

The methodology used was essentially that described by Mejia *et al.* [20]. For the analysis, only the pulp of fresh bell pepper was used. The peppers were chopped into fine pieces. Triplicate samples (10 g) were taken. The 10 g sample was combined with 1 g of magnesium carbonate, 10 g of sodium sulfate and 75 mL of tetrahydrofuran (THF) stabilized with 0.015% of butylated hydroxytoluene (BHT). The mixture obtained was homogenized twice at low speed for 2.5 a 3 min and kept on ice for 3–5 min to cool. The mixture was filtered and the residue was mixed with a second portion of 75 mL of THF and the extraction was repeated. The two filtrates were combined and the total filtrate was flash evaporated to a smaller volume using a Buchi rotary evaporator with temperature of ≤40 °C. The reduced volume obtained was made up to a specific volume of 25 mL with THF to be analyzed by HPLC. Identification and quantification was carried out by HPLC, using a Varian ProStar 320 HPLC system with UV-Vis ProStar 210 detector and a C18-type column (Waters Nova-Pack (Waters Corp., Milford, MA, USA) of 3.9 mm × 15 cm. The solvent system was of acetonitrile/THF/H_2_O (85:12.5:2.5) pumped at a flow rate of 1 mL/min. All runs were at 24 °C and detection at 460 nm. HPLC peaks were identified using retention time comparison with trans-α-carotene (Type V) and β-carotene (Type IV) standards and internal standards. The limit of detection and limit of quantification of the analytical method used for β-carotene quantification were 0.03 and 0.10 μg/mL, respectively.

**Figure 4:**
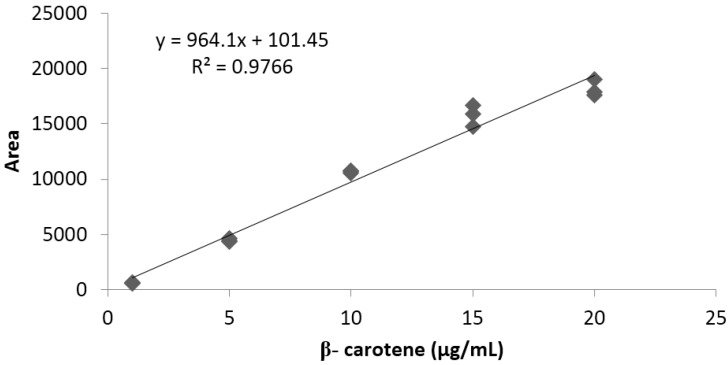
Representative standard curve obtained for β-carotene.

Linearity was determined between 1 and 20 μg/mL, using a standard β-carotene high purity reagent grade, calibrated in triplicate. A typical calibration curve obtained for β-carotene is illustrated in Figure 4.

#### 2.2.5. Antioxidant Capacity

The antioxidant capacity of bell pepper was determined following the technique reported by Zhuang *et al.* [21]. In order to obtain the antioxidant activity, an ethanolic extract was prepared, fresh peppers were ground and triplicate samples (5 g) were taken. The 5 g sample was extracted by stirring with 75 mL 80% ethanol at room temperature for 24 h, and filtered. The filtrates were concentrated using a rotary vacuum evaporator at 40 °C. The resultant extracts were used to determine the antioxidant activities. An aliquot of 0.4 mL of extract was taken and mixed with 2 mL of 0.1 mM DPPH methanol solution. The mixture was kept at room temperature in the dark for 30 min and its absorbance was recorded at 517 nm in a microplate reader.

The DPPH radical-scavenging activity was calculated according to Equation (1):

% scavenging activity = ((A_control_-A_extract_)/A_control_) × 100 (1)

where A_control_ = absorbance of the control, and A_extract_ = absorbance of the extract.

**Figure 5:**
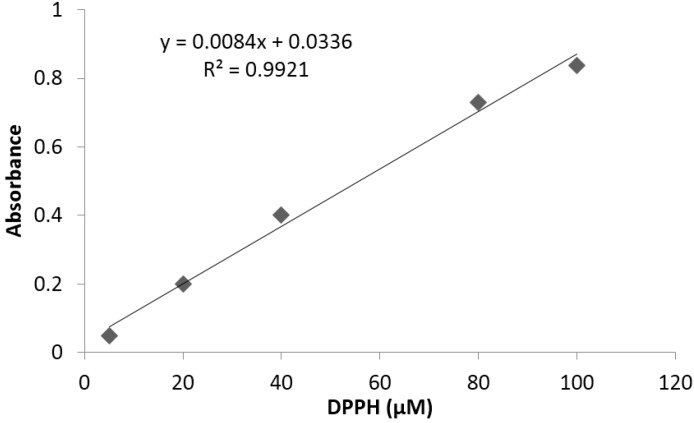
Representative standard curve obtained for antioxidant capacity.

The limit of detection and quantification of the analytical method used for antioxidant capacity determination were 0.6 and 1.85%, respectively. Linearity was determined between 5 and 100 μg/mL, using a standard ascorbic acid (vitamin C) high purity reagent grade, calibrated in triplicate. A typical calibration curve obtained for DPPH is illustrated in Figure 5.

### 2.3. Statistical Analysis

All analyses were performed in triplicated. The results were analyzed by analysis of variance, and a comparison of means was made by Tukey’s test (SAS Inst. Inc., Cary, NC, USA). The means were accepted as significantly different at a 95% confidence interval (*p* ≤ 0.05). In addition, a correlation analysis between the antioxidant capacity and bioactive compounds was conducted.

## 3. Results and Discussion

**Figure 6:**
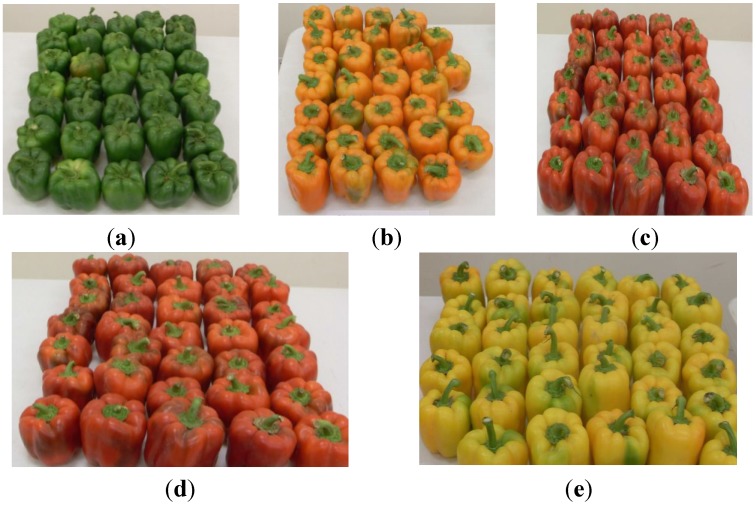
Samples of bell peppers harvested on 3 July 2013 used in the analysis: (**a)** Sweet/Robusto; (**b**) Orangela/Terrano; (**c**) Fascinato/Robusto; (**d**) Fascinato/Terrano; and (**e**) Jeanette/Terrano.

In the present work, the content in bioactive compounds and the antioxidant activity of varieties of grafted bell pepper was characterized. Figure 6 shows the morphology of grafted bell pepper studied, which correspond to the harvest of 3 July 2013.

### 3.1. β-Carotenes

**Figure 7:**
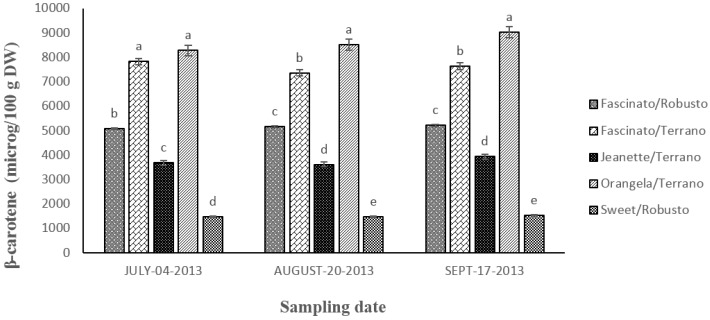
Content in β-carotenes in grafted bell pepper harvested on three different sampling dates. ^a–e^ Different letters indicate statistical difference (*p* ≤ 0.05).

Figure 7 shows the content in β-carotenes found in grafted bell pepper in three sampling dates. The statistical analysis showed differences (*p* ≤ 0.05) between cultivar/rootstock combinations and between sampling dates. On average, a greater content in β-carotenes was found in the peppers harvested on September.

The concentration of this compound diminished as follows: Orangela/Terrano > Fascinato/Terrano > Fascinato/Robusto > Jeanette/Terrano > Sweet/Robusto.

Orangela/Terrano had a 5.8-fold greater content in β-carotenes than Sweet/Robusto, 2.3-fold more than Jeanette/Terrano, 1.7-fold more than Fascinato/Robusto, and 1.1-fold more than Fascinato/Terrano. Orangela/Terrano had an average content in β-carotenes of 8616.19 μg/100 g of dry weight. In all the samples, it presented the highest concentration of all the varieties evaluated. By contrast, Sweet/Robusto registered the lowest concentration, with an average of 1500.2 μg/100 g of dry weight.

The results found in this study were lower than reported by Hallmann and Rembialkowsca [22] in bell pepper varieties Roberta, Spartacus, and Berceo, cultivated both in the conventional system as well as organically in Poland but higher than those found in bell pepper varieties Mazurca, Parker, Torkel, Anupam, Ha-1195, Flamingo, Manhattan and Bomby evaluated by Deepa *et al.* [6].

Orangela/Terrano and Fascinato/Terrano presented a higher quantity of β-carotenes than in the peppers Serrano, Jalapeño, Poblano, and Caribú, while Fascinato/Robusto had a content similar to that found in Serrano pepper [20]. Carotenes are responsible for the yellow, red, and orange colors of fruits and vegetables, and offer beneficial health effects against different diseases such as cardiovascular disease, cancer, and other chronic diseases [23].

According to the results of the present study, the β-carotene content depends strongly on the variety. Thus, in agreement with Marín *et al.* [24], the concentration of this compound also depends strongly on the ripeness of the fruit, being higher in fully ripe fruit (red) than in immature fruits (green). This finding has been reported also by Deli *et al.* [25]. In another study, Blanco-Ríos *et al.* [3] found a higher concentration in peppers of the variety Simpaty (orange), followed by the variety Mazurca (red), then by Taranto (yellow), and finally the lowest content in the cultivar Orion (green), very similar results to those obtained in the present work.

Fascinato/Terrano registered a higher content of β-carotene than Fascinato/Robusto in all the sampling dates, indicating the greater compatibility with the rootstock Terrano than with Robusto. In agreement with Davis *et al.* [26], grafting affects the quality of vegetables and thus careful selection of the combination of scion/rootstock is vital to ensure high fruit quality. There are many reasons why the graft affects fruit quality; the most obvious is the incompatibility between the scions/rootstock, which induces sub-growth or overgrowth of the graft, resulting in a decrease in the flow of water and nutrients through the grafted binding causing wilt.

In agreement with Tundis *et al.* [27], the main reason for studying carotenoids in peppers is due to their bioactive properties, such as their provitamin A activity, antioxidant action, immune modulation and involvement in cell signaling. β-carotene, also, has been extensively investigated as a possible cancer preventive agent; however, the greatest benefits are obtained when these compounds are consumed along with other phytochemicals in whole foods, not from expensive dietary supplements [28].

### 3.2. Vitamin C

**Figure 8:**
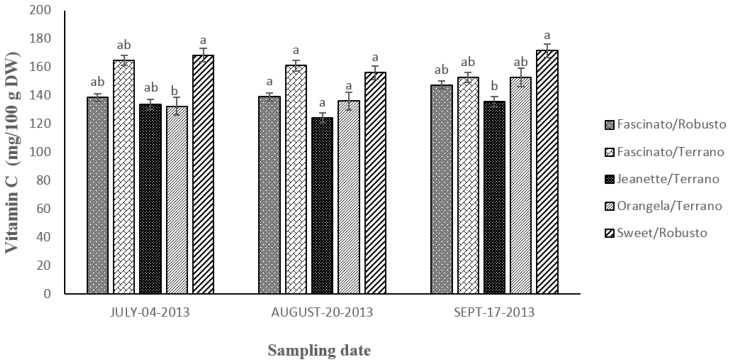
Vitamin C content in grafted bell peppers harvested on three different sampling dates. ^a,b^ Different letters indicate statistical difference (*p* ≤ 0.05).

Figure 8 shows the content in vitamin C found in grafted bell pepper harvested on three sampling dates. The statistical analysis revealed differences (*p* ≤ 0.05) between cultivar/rootstock combinations but not between sampling dates (*p* ≥ 0.05).

On average, the vitamin C content in the cultivar/rootstocks combination analyzed declined in the following order: Sweet/Robusto > Fascinato/Terrano > Fascinto/Robusto > Orangela/Terrano > Jeanette/Terrano.

Sweet/Robusto had 1.3-fold higher content in vitamin C than Jeanette/Terrano, 1.2-fold more than Fascinato/Robusto; 1.16-fold more than Orangela/Terrano, and 1-fold more than Fascinato/Terrano.

Sweet/Robusto registered an average content in vitamin C of 165.56 mg/100 g dry weight, surpassing all the varieties evaluated in the sampling of July and September 2013, while in the August sampling the values were slightly lower than the concentration found in Fascinato/Terrano, although the difference was not statistically significant.

By contrast, Jeanette/Terrano had a lower vitamin C concentration, with an average content of 131.21 mg/100 g dry weight. These figures are lower than those found by Hallmann and Rembialkowska [22] in bell pepper cultivars Roberta, Spartacus and Berceo cultivated in conventional and organic system; and higher than those reported by Ghasemnezhad *et al.* [29] in the bell pepper genotypes Arian, Marona, Zorro, Y-43-09, and Y-43-07 under hydroponic cultivation.

Bell peppers are known to be an excellent source of vitamin C. Data from other studies has shown that the concentration of this vitamin increases with fruit ripeness [30,31]. In this respect, Navarro *et al.* [32] found that the amount of vitamin C incremented from the immature state of the fruit (green) to ripeness (red) with a higher concentration in the transition from green to red. According to Márkus *et al.* [33] vitamin C tended to increase at the onset of ripening, and later decreased with advanced ripening due to its antioxidant role, which increases with the increasing respiration rate in the climacteric fruits. Similar results have been found by Ghasemnezhad *et al.* [29] in the bell pepper cultivars Arian, Marona, Zorro, Y-43-09, and Y-43-07 grown hydroponically.

All the varieties analyzed in the present study are an excellent source of vitamin C, and exceeded the recommended daily dosage (60 mg/100 g) [34]. On average, Sweet/Robusto contributed with 26% of the recommended daily dosage (RDD) and Janette/Terrano with 218%. These results are consistent with other studies reporting that bell peppers contribute from 25% to 461% of the RDD of vitamin C [31,35]. Howard *et al.* [31] reported that the variety Yellow Bell Pepper 47, immature and ripe, contributed with 190 and 225% of the RDD, respectively.

The identification of dietary sources of vitamin C is very important because humans do not have the capability to synthesize vitamin C due to a series of mutations of the gene encoding gulonolactone oxidase which catalyses the last enzymatic step in ascorbate synthesis [36]. Vitamin C is an essential dietary nutrient for a variety of biological functions. Under physiological conditions, it is fundamental in the biosynthesis of collagen and serves in humans also as a co-factor in several important hydroxylation reactions, such as the biosynthesis of catecholamines, l-carnitine, cholesterol, amino acids, and some peptide hormones. In addition, vitamin C may reduce the incidence of most malignancies in humans and positive relationships have been shown between ascorbate supplement use and reduced incidence of Alzheimerʼs disease [37].

Much of the current focus on vitamin C has been on its role as an antioxidant. Antioxidant nutrients are thought to provide a buffer against cell damage that occurs during routine cell functions. Studies that examine the eating habits of large groups of people show that a high intake of vitamin C rich foods may reduce the risk of cancers of the mouth, pharynx, esophagus, stomach, lung, and pancreas. Furthermore, laboratory tests in animals have shown that vitamin C inhibits tumor growth and reduces the genetic damage caused by carcinogens. A potential role for vitamin C in the maintenance of eye health has also been found [38].

Fascinato/Terrano showed higher content of vitamin C than Fascinato/Robusto, showing better compatibility with the commercial rootstock Terrano than with Robusto. Previous studies have indicated that the rootstock Terrano improves the nutritional quality of bell pepper and increases the content of some bioactive compounds including vitamin C [39]. López-Marín *et al.* [40], however, found no differences in the vitamin C content of bell pepper variety Herminio ungrafted and grafted with Terrano. Gisbert *et al.* [12] found no differences, in the content of this vitamin in the pepper hybrids Almuden and Charlot grafted onto the rootstocks Charlot and Foc.

### 3.3. Lycopenes

**Figure 9:**
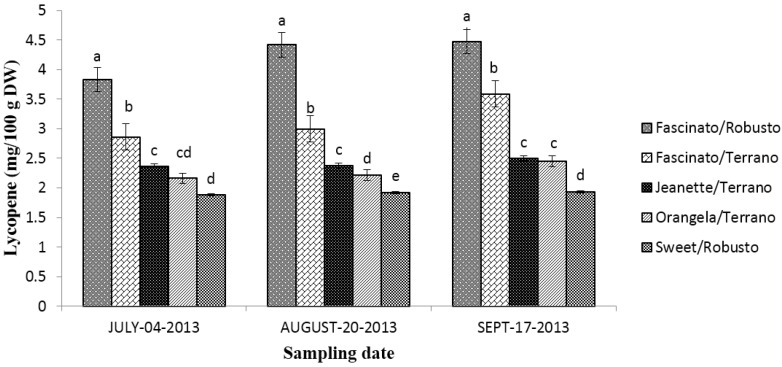
Lycopene content in grafted bell peppers harvested on three different sampling dates. ^a–e^ Different letters indicate statistical difference (*p* ≤ 0.05).

Figure 9 shows the lycopene content in grafted bell peppers sampled on three different dates. The statistical analysis showed differences (*p* ≤ 0.05) between cultivar/rootstock combinations, sampling dates, and the interaction of “cultivar/rootstock combinations x sampling dates”.

The lycopene content in the cultivar/rootstock combinations diminished as follows: Fascinato/Robusto > Fascinato/Terrano > Jeanette/Terrano > Orangela/Terrano > Sweet/Robusto. This pattern held for all three sampling dates.

On average, a higher lycopene concentration was found in the bell peppers sampled in September (*p* ≤ 0.05) than in July or August; the difference between these latter two months was not statistically significant.

Fascinato/Robusto had an average lycopene concentration of 4.23 mg/100 g of dry weight, surpassing all the varieties evaluated, while the variety Sweet/Robusto had the lowest content, averaging 1.91 mg/100 g of dry weight. These results are lower than those reported by Navarro *et al.* [32] in turning and red bell peppers.

Tomato and its products are reportedly the main source of lycopene in the diet. At least 85% of the lycopene that we consume comes from these fruits, and the rest are obtained from other food sources such as apricots, pink grapefruit, watermelon, guava, and papaya [41].

Another important source of lycopene is the red bell pepper [42]. The lycopene values found in the present work were lower than reported by Vincovic-Vrcek *et al.* [43] in tomato varieties Herman, Efialto, and Maxifort grafted to the rootstock Tamaris cultivated in a greenhouse. These authors found no differences between grafted and ungrafted tomatoes. In this context, the bell peppers evaluated in the present work registered a lower lycopene content than watermelon varieties “Black Diamonds” and TriX313 in a state of complete ripeness, evaluated by Perkins-Veazie [44] and lower than Thai watermelon variety Jin-trarah studied by Charoensiri *et al.* [45].

According to Igbokwe *et al.* [4], red peppers have ninefold more lycopene than do green ones. In the present work, Fascinato/Robusto (red) contained twofold more lycopene than Sweet/Robusto (green). The differences in the lycopene levels depend on diverse factors that include variety, sampling method, preparation, method of determination, natural variation among fruits, fertilization, agroclimatic conditions, soil properties, solar radiation, geographic origin, and post-harvest conditions, among others [45].

Interest in carotenoids, particularly lycopene, has grown rapidly owing to studies suggesting a role in human health and disease. Lycopene is not toxic and has antioxidant, anti-inflammatory and chemotherapeutic effects in cardiovascular or neurodegenerative disease and in some cancer [46]. Many of the putative biologic effects and health benefits of lycopene are hypothesized to occur via protection against oxidative damage [47]. Lycopene appears to be the most efficient quencher of singlet oxygen and free radicals among the common carotenoids *in vitro*, this is due to its stereochemical properties. Because lycopene is not converted to vitamin A, it may be entirely available for other properties (e.g., antioxidation) and the lack of the b-ionone ring structure for lycopene may increase its antioxidant activity, this properties are quite different from those of other commonly consumed carotenoids, making it uniquely present in specific subcellular environments [48].

### 3.4. Total Phenols

Among the phytochemical compounds, polyphenols are of particular interest due to their property of scavenging free radicals *in vivo*. Epidemiological studies have shown a possible association between the consumption of polyphenols and a lower risk of coronary disease and cancer [49]. These benefits are due to its high antioxidant activity; antioxidants are hypothesized to play an important role in chronic disease prevention, because they might be able to prevent oxidative damage caused by reactive oxidant species to vital biomolecules such as DNA, lipids and proteins [50].

**Figure 10:**
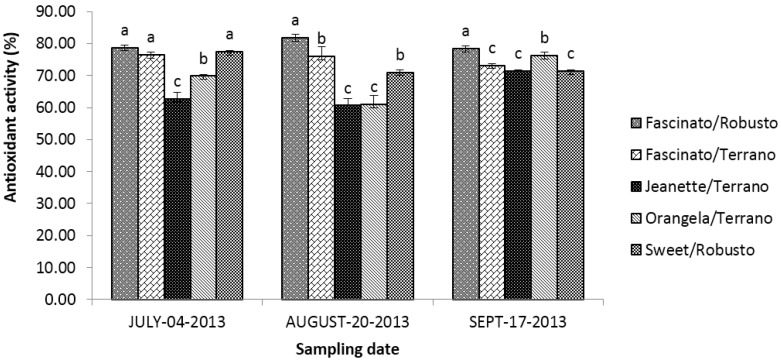
Total phenols content in grafted bell pepper varieties harvested on three different sampling dates. ^a–c^ Different letters indicate statistical difference (*p* ≤ 0.05).

Bell peppers are rich in these types of compounds. Figure 10 presents the phenol content in grafted varieties of bell pepper sampled on three different dates. The statistical analysis indicated a significant difference (*p* ≤ 0.05) between cultivar/rootstock combinations and sampling dates.

The average content in total phenols in cultivar/rootstock combinations diminished as follows: Fascinato/Robusto > Jeanette/Terrano > Fascinato/Terrano > Orangela/Terrano > Sweet/Robusto.

On average, the bell peppers harvested in September had higher (*p* ≤ 0.05) contents in total phenols than in July and August, the difference between these latter two months not being statistically significant.

Fascinato/Robusto had an average concentration in total phenols of 111.26 mg/100 g of dry weight, exceeding all cultivar/rootstock combinations evaluated, while Sweet/Robusto had the lowest content, averaging 70.39 mg/100 g of dry weight. These figures are higher than those given by Navarro *et al.* [32] in turning and red bell peppers and were lower than those given by Zhuang *et al.* [21] in red peppers; these authors reported that the total phenols increased as the peppers advanced in their state of ripeness, and thus the completely red fruits contained significantly higher quantities of this compound than the green fruits. Lee *et al.* [51] also reported an increase in the phenolic content of peppers with maturity. In another study, Hallman and Rembialkowska [22] found that the content in phenolic compounds is influenced also by the crop, as the organic system gives higher values than does the conventional one.

The content in total phenols in the present study depended on the variety and color of the bell pepper fruits. Higher contents in total phenols were found in bell peppers with color than in green ones, values being highest in red, followed by yellow, and then by orange. These results differ from the findings of Blanco-Ríos *et al.* [3], who found that the variety Orion (green) had the highest concentration in this compound, while no differences were detected between the varieties Mazurca (red), Simpaty (orange), and Taranto (yellow). In another study, Sun *et al.* [52] found greater phenolic content in red peppers, followed by orange, yellow, and finally green, as in the present study.

### 3.5. Antioxidant Activity

Among vegetables, bell peppers have been extremely popular for the abundance and types of antioxidants they contain. The phytochemical antioxidants that deserve special mention due to their strong capacity to scavenge free radicals are polyphenols, which are found in high quantities in bell peppers, whose levels vary strongly during growth and ripening [53].

**Figure 11:**
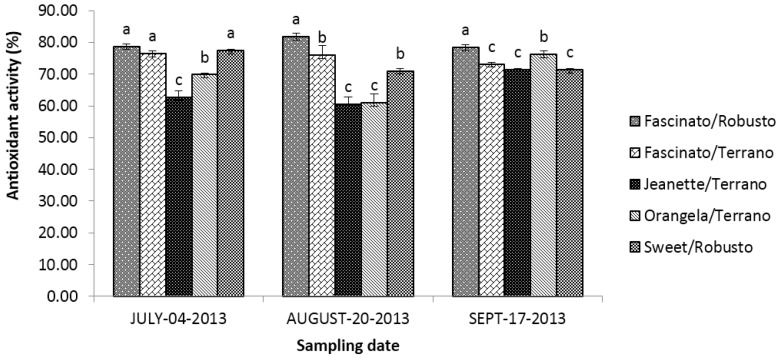
Antioxidant activity of grafted bell pepper varieties harvested in three different sampling dates. ^a–d^ Different letters indicate statistical difference (*p* ≤ 0.05).

Figure 11 presents the antioxidant activity of grafted bell pepper varieties at three different sampling dates. The statistical analysis showed differences (*p* ≤ 0.05) between cultivar/rootstock combinations, sampling dates and the interaction of “cultivar/rootstock combinations *x* sampling dates”.

On average, the bell peppers harvested in September had higher (*p* ≤ 0.05) antioxidant activity than those sampled in July and August. Meanwhile, Deppa *et al.* [6] found no differences in the antioxidant activity of bell peppers between two years of harvest. In the present study, the average antioxidant activity of the cultivar/rootstock combinations diminished as follows: Fascinato/Robusto > Fascinato/Terrano > Sweet/Robusto > Orangela/Terrano > Jeanette/Terrano.

Thus, Fascinato/Robusto had the highest antioxidant activity of all cultivar/rootstock combinations evaluated, with an average of 79.65%, while Jeanette/Terrano presented the lowest activity, with 64.90%. These results are higher than those of Deepa *et al.* [6] in red bell peppers, which were found to have an antioxidant activity of 20%–71.7%; for this range, the lowest concentration was presented by the variety Parker and the highest by the variety Flamingo. Previous studies showed that grafting improved the antioxidant activity of the variety Fascinato but not of Jeanette [39].

Phenolic compounds play a major role in the antioxidant activity in the bell peppers [52]. In the present study, Fascinato/Robusto presented the highest concentration of total phenols, as well as of lycopene in all cultivar/rootstock combinations evaluated, which coincides with a higher antioxidant activity.

The antioxidant activity of bell peppers can also be affected by ripeness. That is, the green fruits have a lower activity than the red ones, perhaps due to differences in the content in carotenoids, phenols, and flavonoids, which contribute significantly to the antioxidant activity and are found in red peppers but are scarce in green ones [52]. Howard *et al.* [30] reported higher antioxidant activity in ripe peppers of the variety Yellow Bell than in the immature ones (42.75% and 66.9%, respectively).

In the present study, the antioxidant activity was significantly affected by the cultivar/rootstock combinations and color. On average, the red bell peppers showed higher activity, followed by green, then orange, and finally yellow, although this depended primarily on the sampling date. The results were similar to those of Blanco-Ríos *et al.* [3], who found higher antioxidant activity in the red peppers (variety Mazurca), followed by green ones (Orion), then yellow ones (Taranto), and finally by orange ones (Simpaty).

### 3.6. Correlations

The antioxidant activity of bell peppers can be attributed also to the content in vitamin C, carotenoids, and capsaicinoids, and therefore it is important to analyze the correlation between the antioxidant activity and the bioactive compounds of bell peppers due to the influence of other soluble compounds in addition to polyphenols, which could affect the total antioxidant capacity [54].

In the present study, the results revealed that in general in all the grafted varieties there was a correlation (*p* ≤ 0.05) between antioxidant activity and the lycopene and vitamin C concentration. In Sweet/Robusto the relation with lycopene was inverse (*r* = −0.78). Jeanette/Terrano was the only cultivar/rootstock combination in which the antioxidant activity was correlated with lycopene, β-carotenes and total phenols; in all cases, the correlation was direct (*r* = 0.85, *r* = 0.81 and *r* = 0.69 respectively). Meanwhile, no correlationsa (*p* ≥ 0.05) was found in Fascinato/Robusto, Orangela/Terrano and Fascinato/Terrano. Zhuang *et al.* [21] found a correlation between the antioxidant activity and the total phenols in bell pepper varieties. Similary, Medina-Juárez *et al.* [55] reported a close correlation between total phenols and antioxidant activity in bells peppers harvested in Zamora, Sonora, México (*r* = 0.91). While, Segura *et al.* [49] did not found correlation between total phenolic compounds and antioxidant capacity in Chiltepin and Habanero peppers, which may indicate that antioxidant capacity could be affected by the presence of phytochemicals such as carotenoids. Hwang *et al.* [56] reported a positive correlation between ascorbic acid, total phenols and antioxidant activity of cooked peppers. Many reports indicate correlation between antioxidant activity and phenolic compounds, of various food samples, for example, Yao *et al.* [57] reported a significant positive correlation between the antioxidant activity and the contents of total flavonoids and total phenols in celery. Campos *et al.* [58] found high correlations between antioxidant activities and total phenolic contents in mashua and oca tubers. It is well known that phenolic compounds exert antioxidant activity in biological systems. To estimate the inhibitory capacity of these compounds against reactive oxygen species, data correlating antioxidant activity and phenolic concentration are commonly reported [59].

## 4. Conclusions

The results of the present study reveal differences in the content of bioactive compounds and antioxidant activity among the grafted varieties of bell pepper. Fascinato/Robusto presented the highest concentrations of lycopenes and total phenols as well as the greatest antioxidant activity of all/cultivar/rootstock combinations evaluated. Meanwhile, Sweet/Robusto had the highest content in vitamin C and Orangela Terrano in β-carotenes. Furthermore, the best sampling date to have the highest content in bioactive compounds and strongest antioxidant activity proved to be in September. On the other hand, Fascinato/Terrano was a good source of bioactive compounds and antioxidant activity, as it was consistently cultivar/rootstock combination with the second-highest quantity of these compounds.
